# Interferon-γ Release Assay in the Assessment of Cellular Immunity—A Single-Centre Experience with mRNA SARS-CoV-2 Vaccine in Patients with Juvenile Idiopathic Arthritis

**DOI:** 10.3390/jcm13092523

**Published:** 2024-04-25

**Authors:** Katarzyna Kapten, Krzysztof Orczyk, Anna Maeser, Elzbieta Smolewska

**Affiliations:** 1 Department of Pediatric Cardiology and Rheumatology, Medical University of Lodz, 91-738 Lodz, Poland; katarzyna.kapten@umed.lodz.pl; 2 Department of Pediatric Infectious Diseases, Medical University of Lodz, 91-347 Lodz, Poland; krzysztof.orczyk@umed.lodz.pl; 3 Department of Pediatric Cardiology and Rheumatology, Central Teaching Hospital of Medical University of Lodz, 91-738 Lodz, Poland; annamaeser1@gmail.com

**Keywords:** SARS-CoV-2, mRNA vaccine, cellular immunity, juvenile idiopathic arthritis

## Abstract

**Background**: As the SARS-CoV-2 virus remains one of the main causes of severe respiratory system infections, the Food and Drug Administration strongly advises the continuation of current vaccination programs, including the distribution of updated boosters, especially in high-risk groups of patients. Therefore, there is an unceasing need for further research on the safety and, no less importantly, the clinical effectivity of the vaccines, with an extra focus on cohorts of patients with underlying health problems. This study aimed to assess the efficacy of the SARS-CoV-2 vaccine in possibly immunocompromised children with rheumatic disease while utilizing the interferon-gamma release assay (IGRA) as a marker for COVID-19 immunity in the study follow-up. **Methods**: This prospective study was performed in a group of 55 pediatric patients diagnosed with juvenile idiopathic arthritis. Eight participants were immunized with the Comirnaty mRNA vaccine before the research commenced, while the rest of the group (*n* = 47) had not been vaccinated against SARS-CoV-2. At the study baseline, the cellular response to the virus antigen was measured using a specific quantitative IGRA in whole blood; subsequently, the anti-SARS-CoV-2 test was performed, marking the antibodies’ levels in serum. Around four months after the enrollment of the last patient in the study, a follow-up survey regarding the events of COVID-19 infection within the cohort was conducted. **Results**: The study confirmed that all the vaccinated children developed specific T-cell (*p* = 0.0016) and humoral (*p* = 0.001 for IgA antibodies, *p* = 0.008 for IgG antibodies) responses to the inoculation, including those receiving biological treatment and those on conventional disease-modifying anti-rheumatic drugs. The study also showed the different patterns of immunity elicited both after infection and post-vaccination, with higher levels of antibodies and T-cell response after inoculation than after natural exposure to the pathogen. According to the follow-up survey, six children developed PCR-confirmed SARS-CoV-2 infection, whereas the additional 10 patients admitted to having COVID-like symptoms with no laboratory verification. **Conclusions**: SARS-CoV-2 vaccinations elicit valid immune responses in pediatric rheumatic patients. Including the assessment of T-cell immunity in the evaluation of inoculation-induced immunization can enhance the accuracy of sole humoral response assays.

## 1. Introduction

As of July 2023, more than 70% of the world population had received at least one dose of the SARS-CoV-2 vaccine, which accounts for over 13 billion doses administered globally and more than 40 thousand shots administered every single day [1]. A variety of advanced vaccine candidates, including i. a. mRNA, inactivated virus, protein subunit and virus-like particles, entered different phases of clinical trials [2], while more than 20 have met approval to be used [3]. The current guidelines of the United States Food and Drug Administration (FDA) for fall 2023 advise that everyone, including children older than six months, should receive at least one dose of the Pfizer-BioNTech or Moderna COVID-19 vaccine updated with an XBB-lineage of the Omicron variant [4]. Moreover, moderately and severely immunocompromised patients may require additional boosters [5]. That being said, there is an urgent and unfaltering need for continuous monitoring of both the safety and efficacy of vaccines against SARS-CoV-2.

There is still a lack of consensus regarding the risk of more severe or fatal COVID-19 infection in patients suffering from autoimmune and inflammatory disorders, including rheumatic diseases (RDs). While initial studies suggested a similar or just slightly higher risk of contracting the virus or worse prognosis for infection in patients with RDs compared to healthy individuals [6,7], a more recent and extensive meta-analysis states that RDs predispose to substantially higher rates of SARS-CoV-2 infection and increased mortality [8]. Notably, most of the data available are obtained from the adult population, leaving a knowledge gap in the management of pediatric patients. While the studies that evaluated the course of COVID-19 in children with RDs did not prove a significantly higher risk of poor disease outcomes compared to the previously healthy population, the infection can still lead to a flare of the underlying condition and a need for therapy escalation. In some cases, it can result in a reduced health-related quality of life [9,10]. Thus, pediatric patients with RDs or other autoimmune diseases seem to represent a special group of interest in which protective measures taken to prevent the severe course of COVID-19 should remain significant.

After a successful trial of the BNT162b2 mRNA vaccine in adults, it was also approved for the inoculation of children between the ages of 12 and 18. Regarding the safety of vaccination in the population suffering from RDs, it may be important to acknowledge the dependencies between the SARS-CoV-2 vaccine and the onset of autoimmune diseases mentioned in several studies. In their extensive systematic reviews of new cases of arthritis or the worsening of the already existing condition as a result of the SARS-CoV-2 vaccine, researchers stressed the need for awareness of joint-related inoculation side effects. However, the lack of data from well-controlled trials questions the significance of these findings. Additionally, in most of the patients, the clinical symptoms subsided after the use of nonsteroidal anti-inflammatory drugs or glucocorticoids without the need to introduce disease-modifying antirheumatic drugs (DMARDs) [11]. A similar response to steroid therapy, with a tendency to resolve spontaneously in some cases, was observed in SARS-CoV-2 vaccine-induced vasculitides [12]. However, there are also reports of more severe, life-threatening cases of autoimmune and autoinflammatory syndromes after the new vaccination, including dermatomyositis complicated by lung disease [13] and adult-onset Still disease [14]. Markedly, these complications were predominantly found in adults, seemingly sparing the pediatric population.

The safety and presence of humoral response to the vaccination in the cohort of adolescents with RDs have been confirmed in the prospective studies [15,16]. However, while the most accurate assessment of inoculation efficacy should be performed in randomized control trials (RCTs) with the evaluation of mild to severe infections, the assessment may be problematic in trials with smaller sample sizes [17,18]. Therefore, the majority of available data on the assessment of vaccine potency in children with RDs are based primarily on the sole analysis of antibody titers.

**Figure 1:**
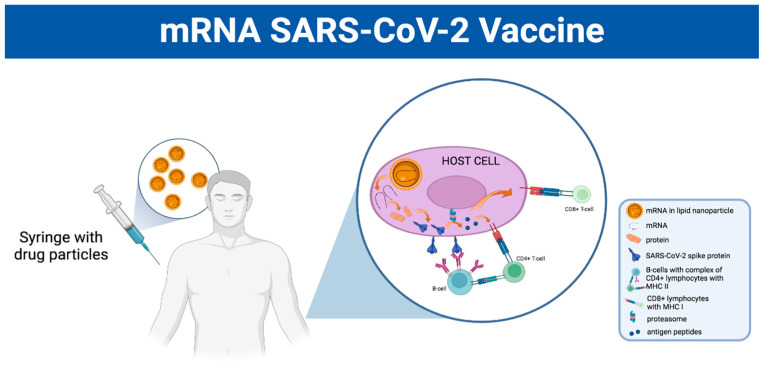
Mechanism of messenger ribonucleic acid (mRNA) vaccine. The mRNA molecules encapsulated in lipid nanoparticles facilitating the release of mRNA into the cells are injected into the body. Once the mRNA molecules are inside the cytosol of the host cell, they are translated into viral protein to elicit a specific immune response. Once inside host cells, the mRNA is translated into SARS-CoV-2 spike protein, which is expressed on the surface of the host cells and is exposed to the extracellular space. The transient expression of this spike antigen induces neutralizing antibodies and cellular immune responses against it. After proteasomal processing, antigen peptides join the major histocompatibility complex class I molecule (MHC I) and are transferred to the antigen-presenting cell surface, activating CD8+ T cells. Protein antigens can activate CD4+ T cells via the MHC Class II presentation pathway.

However, during the pandemic, numerous studies confirmed the utility of marking cellular immunity after COVID-19 infection or inoculation. The currently available mRNA vaccines are found to elicit long-lasting T-cell responses across variants of the virus, detectable even after the decrease in antibody titers [19]. Interestingly, with the new mutations of COVID-19, like Omicron, it was suggested that the T-cell response was retained in vaccinated individuals, even with the lack of neutralizing antibodies [20]. The SARS-CoV-2 interferon-gamma release assay (IGRA), which was previously used mainly in the diagnostics of tuberculosis [21], is a useful tool for the assessment of immune responses after inoculation. The test measures the levels of interferon gamma (IFN-γ) secreted by T-helper 1 and T-cytotoxic cells that were previously primed and activated by SARS-CoV-2-specific S protein produced upon vaccination [22]. The authors present the more detailed mechanism of the mRNA vaccine in Figure 1. Moreover, the assessment of vaccine-elicited cellular response has been successfully applied to cohorts of immunocompromised individuals, including patients suffering from RDs [23,24]. IGRA has already been utilized in the monitoring of cellular response and therefore the efficacy of vaccination in pediatric patients suffering from inflammatory bowel disease [25].

The current research aimed to conduct a comprehensive assessment of both humoral and cellular immunity mounted by the SARS-CoV-2 mRNA vaccine in patients with JIA and to assess IGRA as a marker of immunity against COVID-19 in the study follow-up.

## 2. Materials and Methods

The design of this prospective study included the assessment of humoral and cellular responses to SARS-CoV-2 in a group of vaccinated and unvaccinated pediatric patients with JIA. After the enrollment of all the participants, a follow-up survey regarding breakthrough COVID-19 cases was conducted.

The research was performed in a cohort of 55 children with juvenile idiopathic arthritis (JIA) during their hospitalization in the Department of Pediatric Cardiology and Rheumatology, Medical University of Lodz, Poland, between June 2021 and February 2023 with the follow-up survey 2 years after the recruitment of the first patient and 4 months after including the last participant. All the patients were diagnosed with JIA according to the International League of Associations for Rheumatology (ILAR) classification criteria [26], with onset before the 16th birthday and arthritis persisting for at least 6 weeks. Among the causes of hospitalization were a newly diagnosed JIA, a flare of the disease demanding modification of the treatment, or routine day-case hospitalizations that are a part of biological agent protocols. Eight children within the study group received the Comirnaty mRNA vaccine between 1 and 18 months prior to their enrollment in the study, whereas the rest of the group (*n* = 47) had not been vaccinated against SARS-CoV-2 when the tests were performed. Patients who qualified for the study had both positive and negative histories of COVID-19. The inclusion criteria for the study were a confirmed diagnosis of JIA and a maximum age of 16 years on the day of recruitment. The cohort consisted of both newly diagnosed cases (*n* = 15) and patients with years-long treatments; participants were selected consecutively. None of the patients were in a flare of the rheumatic process severe enough to require high doses of steroids. The results of the follow-up questionnaire were acquired from 53 out of the 55 parents of the patients included in the research. The questionnaire was designed to include all the participants, although two patients could not be reached by phone and were therefore not considered in this final part of the research.

The quantitative variables measured in this study were the cellular and humoral responses to SARS-CoV-2 antigens. The endpoint of the study in the follow-up was COVID-19 (whether confirmed or assumed). While the cohort of the study was not homogenous regarding the age of the children, social status and the received immunomodulating treatment, the study was conducted during the peak of the pandemic in Poland, so exposure to the virus should remain fairly comparable for all the participants. The specific T-cell response to SARS-CoV-2 antigens was measured using quantitative IGRA in whole blood with a Quan-T-Cell SARS-CoV-2 EUROIMMUN assay. Blood samples with 1.5 mL nominal volume were drawn simultaneously with routine laboratory tests during the patients’ hospitalization. The heparinized blood was then incubated in a set of three tubes: (1) IGRA BLANK with no activating components for the individual’s IFN-γ background; (2) IGRA TUBE for specific T-cell stimulation by SARS-CoV-2 antigen spike protein; and (3) IGRA STIM for unspecific T-cell stimulation with mitogen for determining stimulation ability. After the removal of cells during centrifugation, the obtained plasma was analyzed by a quantitative enzyme-linked immunosorbent assay (ELISA) to determine the concentration of released IFN-γ. Additionally, the anti-SARS-CoV-2 ELISA was performed in all patients to mark the levels of IgA, IgM and IgG antibodies. Both IGRA and ELISA assays were performed using the samples gathered at the same time point during the initial enrollment of each participant in the study.

During the follow-up survey, the parents of the children who participated in the study were asked questions regarding confirmed or suspected SARS-CoV-2 infection in the months following the trial. To keep the follow-up survey unbiased, it was conducted by a doctor not previously involved in the study and with no access to the acquired data. The achieved sample size was the result of a two-year timeframe established by the authors at the beginning of the research.

Group comparisons were performed using the Mann–Whitney U test. *p* values below 0.05 were considered significant. To calculate the most accurate cutoff value for IGRA, the authors utilized the Youden index. The sensitivity and specificity of IGRA as a marker for individuals’ susceptibility to SARS-CoV-2 infection were analyzed using the receiver operating characteristic (ROC) curve. All statistical calculations were performed using Statistica 13.1 software (Statsoft Polska, Krakow, Poland). The reporting in this study conforms to STROBE [27].

The study was approved by the local Bioethics Committee, with approval number RNN/117/21/KE. All the diagnostic tools were ordered from EUROIMMUN POLSKA, Wroclaw, Poland. 

## 3. Results

The general characteristics of the study group are presented in Table 1. Patient age, sex, subtype of JIA (oligoarthritis, polyarthritis or systemic-onset arthritis) and time from the initial diagnosis to study onset or rheumatic disease flare were all found to have no statistical significance for the patients’ immune responses.

Participants in the study received various immunomodulating treatments, including biologic and oral disease-modifying antirheumatic drugs; a few children were on moderate or small doses of prednisone. Among the vaccinated individuals, three were receiving biological treatment with tumor necrosis factor (TNF) inhibitor, four were treated with methotrexate, two with hydroxychloroquine and one with azathioprine.

**Figure 2:**
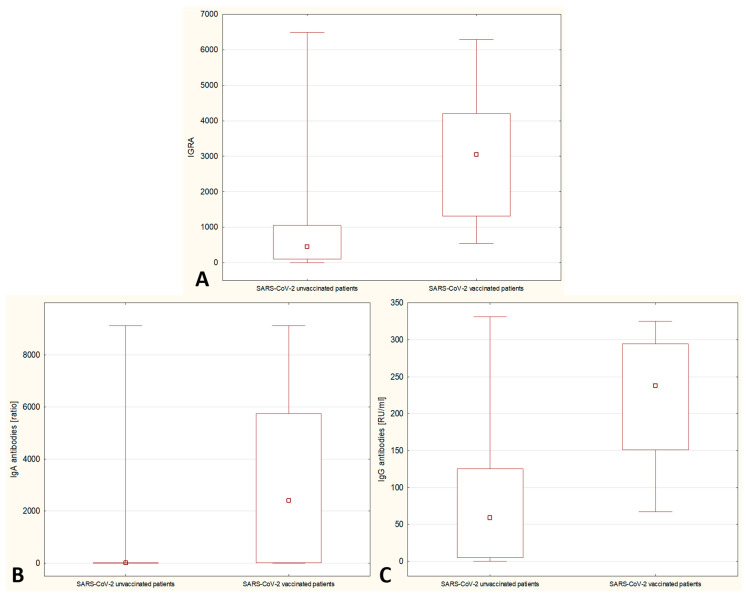
(**A**) Association between Interferon-γ Release Assay (IGRA) and SARS-CoV-2 vaccination. (**B**) Dependency between IgA antibodies and SARS-CoV-2 vaccination. (**C**) Dependency between IgG antibodies and SARS-CoV-2 vaccination.

**Figure 3:**
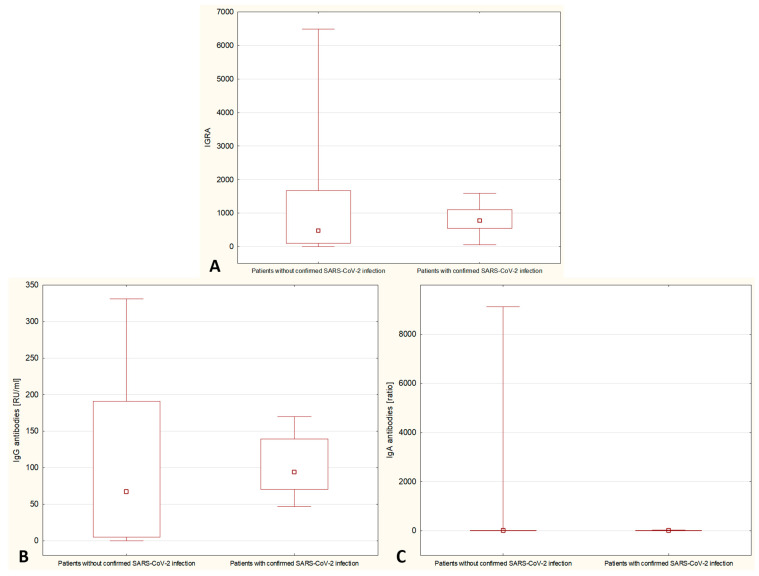
(**A**) Association between Interferon-γ Release Assay (IGRA) and postinfection natural immunity. (**B**) Dependency between IgA antibodies and postinfection natural immunity. (**C**) Dependency between IgG antibodies and postinfection natural immunity.

The research confirmed that all the vaccinated children developed specific T-cell responses measured by IGRA (*p* = 0.0016) (Figure 2A) and humoral responses assessed by antibody titers, significant in IgA (*p* = 0.001) (Figure 2B) and IgG (*p* = 0.008) class (Figure 2C). No dependencies with IgM antibody levels were found. When analyzing and comparing humoral and cellular immunity after vaccination with the naturally induced immune responses, there was a notable difference between the immunity elicited by vaccination and post-exposure immunity. The study indicated higher levels of IGRA (Figure 3A) and higher titers of IgG (Figure 3B) and IgA (Figure 3C) antibodies after inoculation in comparison with natural exposure to the virus.

**Scheme 1:**
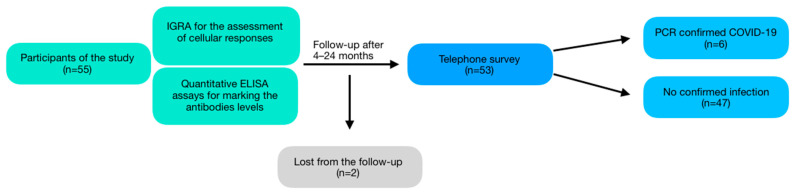
A flow diagram showing the steps of the study and the results of the follow-up survey. IGRA-interferon-gamma release assay, ELISA-enzyme-linked immunosorbent assay, PCR-polymerase chain reaction.

**Figure 4:**
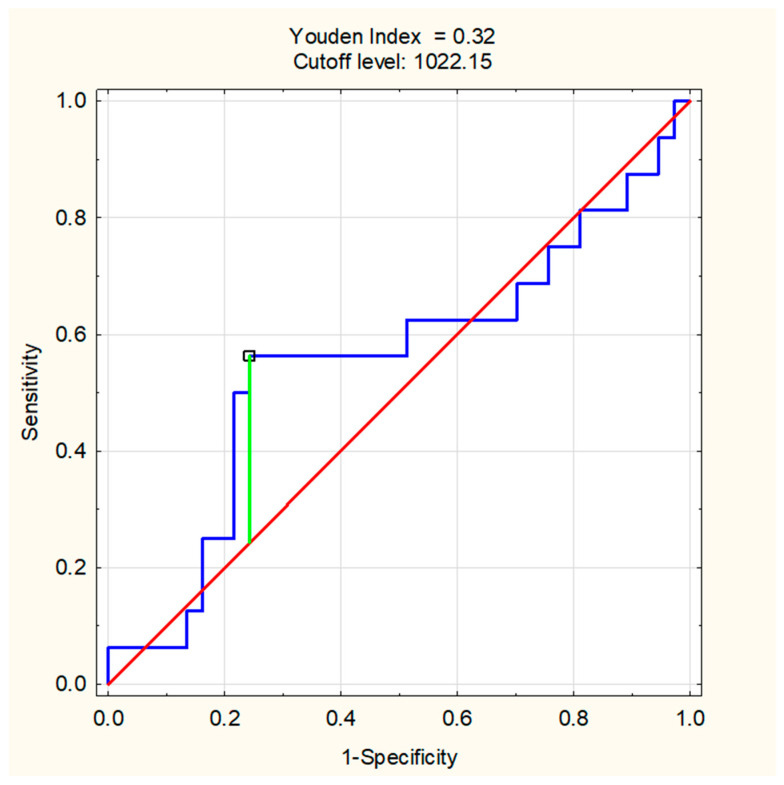
The receiver operating characteristic curve and the cutoff value for a positive IGRA result.

**Figure 5:**
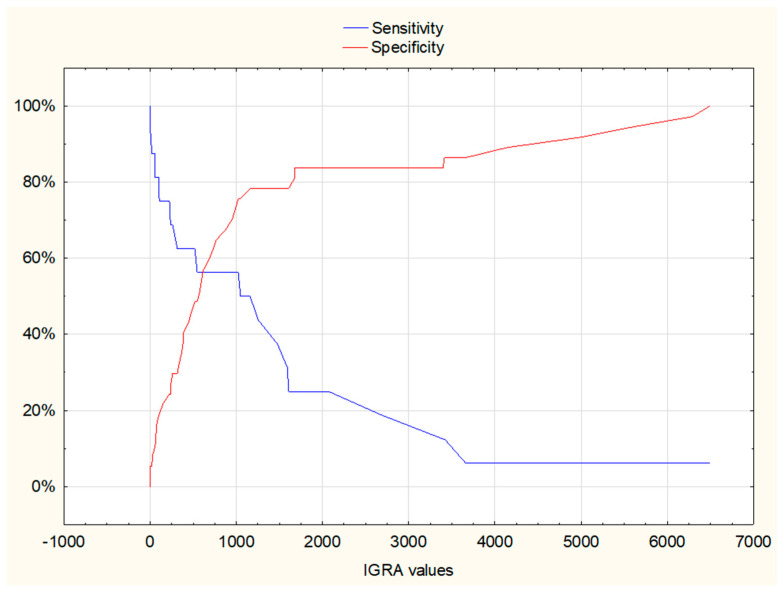
The specificity and sensitivity of IGRA in terms of assessing a patient’s susceptibility to SARS-CoV-2 infection.

Details of the survey are presented in Table 2. Among the participants, 37 patients denied contracting SARS-CoV-2 in the months following their participation in the study. Only six children had SARS-CoV-2 infection confirmed by PCR, while 10 participants admitted to having COVID-like symptoms with no PCR confirmation (Scheme 1). None of the cases of SARS-CoV-2 within the study group were severe. As there is no well-established cutoff value for IGRA, the authors attempted to calculate one utilizing the follow-up survey results. IGRA showed higher specificity and sensitivity when the clinical symptoms were supplemented with laboratory confirmation. The proposed cutoff value for positive IGRA was 1022.15 (Figure 4), with 60% sensitivity and 80% specificity, as calculated with the ROC curve (Figure 5). The results obtained after the follow-up survey indicated that, among the vaccinated patients, significantly lower IgA (*p* = 0.0102), IgG (*p* = 0.058), and IgG NCP (*p* = 0.0029) antibody titers were found in participants with breakthrough SARS-CoV-2 infections.

## 4. Discussion

As far as the main goals of the current research are concerned, the study confirmed the validity of SARS-CoV-2 vaccination programs in the cohort of pediatric patients with RDs. All the participants undergoing treatment protocols and other variables, regardless of the type of JIA, elicited both humoral and cellular responses to the inoculation. Moreover, the application of IGRA to marking T-cell immunity proves to be a viable way to mark individual immunity. The follow-up of the participants confirmed a low rate of breakthrough infections among children with JIA.

It has already been proven in a few sources that the assessment of cellular responses after SARS-CoV-2 infection or vaccination provides a more comprehensive evaluation of individual immunity than the sole use of serological testing [28,29,30]. While different branches of adaptive immunity coordinate to maintain optimal protection against pathogens, T-cells play a key role in controlling the developing infection by modulating disease severity and in creating long-term memory pools that are believed to decline at a slower rate than antibody titers [30,31]. A recent study confirmed a positive association between SARS-CoV-2 specific T-cell responses and antibody titers after inoculation. These results are concordant with multiple studies [30,31,32,33], including one by Agrati et al. [34], who evaluated the elicited immunity to the second dose of the mRNA vaccine, early after the shot and 12 weeks later, noting the decrease in antibody titers while the T-cell memory persisted. Still, the study showed dependencies between cellular and humoral responses, suggesting the perseverance of coordinated immunity. The diversity of IGRA methodology results in difficulties in comparing results between studies. The standardized cutoff level would facilitate the collection of all the results for future meta-analysis. The cutoff values proposed in the current study are considerably higher than in most of the publications, where they were established at around 200 mIU/mL [35,36,37]. According to Lledó et al. [38], cellular responses to SARS-CoV-2 antigens were comparable between RD patients and healthy controls; they stated that neither the disease nor RD therapies should affect individual adaptive immune responses. However, there are some conflicting data on adaptive immunity elicited in children compared to that in adults. Some studies evaluating the immune reaction to SARS-CoV-2 infection in the pediatric population have stated that the specific T-cell responses were comparable between adolescents and adults [39]. However, multiple studies regarding immune reactions to infection or vaccination have identified age as a factor significantly associated with the magnitude of cellular responses to SARS-CoV-2 antigens [40,41,42].

According to previous research on immunological responses to the SARS-CoV-2 vaccine in a group of individuals suffering from autoimmune diseases, the patients did develop humoral response to the inoculation. However, when compared to the healthy controls, the immune reactions were considerably delayed or reduced [43,44]. Notably, in accordance with Simon et al., the reduced effect of the vaccine resulted from the underlying autoimmune disease and its impact on individuals’ immune reactions rather than from the immunomodulating treatment [44]. These results are concordant with the data that were acquired during the current study, as none of the treatments that the patients were receiving proved to affect their immune reactions to the inoculation. However, it should be stressed that this is not always true regarding some specific treatments used in RDs. As seen in the study by Oyaert et al., in which around half of the patients with RDs failed to evoke a humoral response and had lower cellular responses to the vaccine, all the patients were receiving immunosuppressive treatment with rituximab [45]. The current recommendations concerning the management of patients with RDs, including vaccination programs in both adults [6] and children [15,16,17], are based mainly on the antibody-eliciting potential. There is scarce data regarding cellular responses in patients with RDs after vaccination. Nevertheless, in a study by Mahil et al., conducted in a group of patients suffering from psoriasis and receiving immunomodulating treatment (MTX and biological agents), all subjects elicited a humoral response to the vaccine, although some did not have detectable T-cell responses even after the second dose [23]. The potency of existing antibodies and their clinical impact on undetectable cellular responses remains to be further explored.

However compelling the data on elicited humoral and cellular immunity may be, the clinical implications of the obtained results need to remain a priority. In a study by Calcoen et al. [46], even the slight decrease in immune response to SARS-CoV-2 three months after vaccination co-occurred with a high incidence rate of symptomatic breakthrough infections. Additionally, Vogrig et al. [47] conducted a prospective study on a group of 80 vaccinated individuals and reported that both the decreased IGRA response and, to a smaller extent, the humoral response were associated with the higher rate of reinfections during the follow-up period. To conclude, both methods can be valid measures for the prediction of breakthrough infections. A search for corresponding data in the cohort of patients with autoimmune diseases on immunomodulating therapies reveals a certain knowledge gap. Nevertheless, a study by Ahmed et al. [48] confirmed that the SARS-CoV-2 infections occurring after vaccination in patients with RDs were associated with COVID-19 seronegativity. Unfortunately, cellular responses were not taken into account in this paper. The results of the follow-up conducted in the recent study align with those results, as the lower IgA and IgG titers correlated with an occurrence of breakthrough infections after inoculation. Nonetheless, due to the small sample size of the vaccinated children included in the study, these data should be considered more as a trend.

The question of the differences between the immune responses to the SARS-CoV-2 vaccine, in comparison to the naturally acquired immunity, has been a point of interest in multiple studies. Amanat et al. analyzed naive individuals after inoculation with the SARS-CoV-2 mRNA vaccine and found that while antibody responses to the vaccine were robust and even exceeded those seen after natural infection, the majority of vaccine-induced antibodies did not have neutralizing activity [49]. These conclusions should be taken into account when evaluating the results acquired during the current study, where inoculation leads to both higher antibody responses and levels of IGRA than natural infection. Moreover, in a study utilizing single-cell mRNA sequencing to compare similar cohorts of patients, the authors noted the expansion of CD8+ T-cell clones in more distinct clusters induced by natural infection compared to those induced by vaccination, meaning that they were more likely to recognize a broader spectrum of viral antigens, thus indicating the superiority of natural immunization [50].

We acknowledge that our study has some limitations, mostly due to the small sample size of patients who received the mRNA vaccine and the lack of a control group consisting of healthy individuals. Differences in time intervals between the inoculation and sample taking between the study participants may be another effect modifier. Additionally, the results obtained from the survey, for the most part, relied on the parents’ evaluations of their children’s symptoms, which may be highly biased.

To expand and add value to our observations, adding a control group of children without JIA in future research could provide interesting and reliable results regarding the differences in the immunity elicited in patients with RDs and healthy children. A larger cohort study with a comparison group would be highly valuable for future guidelines regarding COVID-19 vaccine programs in this specific cohort of patients. Additionally, the results obtained from the survey, for the most part, relied on the parents’ evaluations of their children’s symptoms, which may be highly biased. A more standardized follow-up of vaccinated children with JIA may provide essential knowledge of the clinical outcomes of inoculations among immunocompromised patients.

## 5. Conclusions

SARS-CoV-2 mRNA vaccine may successfully elicit humoral and cellular immune responses in children with JIA, including patients receiving biological treatment and/or conventional DMARDs. IGRA assays for the evaluation of T-cell responses to inoculation may add value to quantitative ELISA antibody tests. Moreover, the study follow-up indicates a low rate of breakthrough SARS-CoV-2 infections, even in possibly immunocompromised children. The study postulates that pediatric patients with RDs develop substantial immune responses to the SARS-CoV-2 mRNA vaccine, regardless of the disease-modifying therapies they are receiving. Larger cohort studies are needed to establish comprehensive guidelines regarding SARS-CoV-2 inoculation programs in children with RDs.

## Figures and Tables

**Table 1:** General characteristics of the study group.

	Total Number of Patients	Received SARS-CoV-2 Vaccine
	*n* = 55	*n* = 8
Male/Female	14/41	2/6
Age on examination (years)	10.31 ± 4.16	13.75 ± 1.47
Type of JIA:		
systemic	6	2
oligoarticular	29	4
polyarticular	20	2
Newly diagnosed JIA	15	2
Flare of JIA	8	0
History of confirmed SARS-CoV-2 infection	8	0
Treatment regime:		
Biological agents specifically:	22	3
adalimumab	13	3
tocilizumab	6	0
etanercept	2	0
baricitinib	1	0
Methotrexate	32	4
Sulfasalazine	8	0
Hydroxychloroquine	7	2
Cyclosporine	3	0
Azathioprine	1	1
Glucocorticoids	4	0

Values presented as mean ± standard deviation (SD), JIA—Juvenile Idiopathic Arthritis.

**Table 2:** A follow-up telephone survey concerning SARS-CoV-2 infection events.

Questions	Did Your Child Have Confirmed SARS-CoV-2 Infection or COVID-like Symptoms since Being Included in the Clinical Trial?
Answers:	Number of patients
No	37
Possible, present some of COVID-like symptoms, no PCR confirmation	8
Highly possible, many COVID-like symptoms, no PCR confirmation	2
Yes, infection confirmed in PCR	6
Did not answer the phone	2

Question and answers have been translated into English. PCR-polymerase chain reaction.

## Data Availability

The data used to support the findings of this study are included in the article. The supplementary data are available from the corresponding author upon request.
